# Development of ganciclovir as an anti-human cytomegalovirus drug: historical perspectives and future prospects

**DOI:** 10.1099/jgv.0.002310

**Published:** 2026-08-05

**Authors:** Natalia Garcia del Valle, Blair L. Strang

**Affiliations:** 1 Infection and Immunity Research Institute, City St George’s, University of London, London, UK

**Keywords:** aciclovir, congenital, ganciclovir, human cytomegalovirus, phosphorylation

## Abstract

Human cytomegalovirus (HCMV) infection is widespread within the global human population and is a major cause of human morbidity and mortality. As yet, there is no widely available anti-HCMV vaccine strategy. Therefore, treatment of HCMV disease relies on anti-HCMV drugs, principally the nucleoside analogue pro-drug ganciclovir (GCV). While it is over 40 years since the discovery of GCV as an anti-HCMV compound, the use of GCV in humans is still developing, for example in the treatment of congenital HCMV infection. Here, we provide an overview of often overlooked virological laboratory studies underpinning GCV development. We highlight long-standing, but rarely examined, data on adverse effects that is the basis for caution in using GCV during pregnancy or in children. This includes discussion of recent, contrasting data, suggesting that GCV can be further developed to treat congenital HCMV infection. To better understand how adverse effects may come about, we also review the viral and cellular factors required for GCV function in HCMV-infected and uninfected cells. This leads to a discussion of how adverse effects of GCV may or may not be avoided and future directions involving the use of GCV. Overall, these topics highlight how much molecular information remains to be understood about one of the oldest and most widely used anti-HCMV drugs in humans.

## Introduction

Human cytomegalovirus (HCMV) is a herpesvirus found throughout human populations. In immunocompetent individuals, HCMV disease is largely asymptomatic. However, HCMV severely affects immunocompromised individuals. In adults, these individuals are mostly transplant recipients and those with AIDS [1]. In early life, HCMV infection can occur in the foetus, infants and newborns [1–3]. Congenital HCMV infection is a leading non-genetic cause of permanent neurodevelopmental disabilities [1, 3]. Presently, there is no widely available vaccine strategy to combat either HCMV infection or disease. However, several promising strategies are in development, which have been expertly reviewed elsewhere [1, 4]. Historically, treatment of HCMV disease largely relies on one antiviral drug, the nucleoside analogue ganciclovir (GCV) [5–8]. It is now over 40 years since the initial descriptions of GCV as an anti-HCMV compound, but the use of GCV against HCMV is still developing. Here, we present a review of GCV development as an antiviral drug. This historical perspective highlights often overlooked works, including data on GCV use in animal models and its adverse effects. Importantly, discussion of how GCV was developed for use today promotes discussion of its future use. Future use depends upon understanding the molecular basis of GCV function within the cell, as this can be the cause of adverse effects. As we demonstrate, although recent advances in understanding GCV interaction with the virus and the cell have been made, considerable information on the molecular biology of these interactions is still to be uncovered.

## Identification and development of GCV as an anti-HCMV drug

By the late 1970s, human herpes viruses, including HCMV, were recognized as the cause of a spectrum of human disease. Therapeutic development against HCMV then focused on agents such as adenine arabinoside [9], which had shown promise in clinical trials as treatments for herpes simplex virus (HSV)- and varicella-zoster virus (VZV)-associated disease. However, these interventions were associated with considerable treatment failure, as well as dose-limiting toxicities. The AIDS epidemic in the 1980s highlighted the morbidity and mortality that could be associated with HCMV disease and brought widespread attention to HCMV [10, 11]. Unlike HSV, HCMV infections of AIDS patients and others lacked effective antiviral treatment. Therefore, the development of anti-HCMV drugs, such as GCV, was driven by the then urgent necessity to identify therapeutic agents able to inhibit HCMV replication and/or disease.

Within the 1970s/1980s time period, it had been established that the guanine analogue aciclovir (ACV) had strong anti-HSV activity [12–14] as it was understood that essential to the anti-HSV activity of ACV was the ability of an HSV thymidine kinase (TK) enzyme to participate with cellular enzymes in the conversion of the nucleoside pro-drug form of ACV to the triphosphate (TP) nucleotide analogue drug form of ACV, ACV-TP [12]. ACV-TP inhibited HSV DNA replication once incorporated into nascent HSV DNA by the HSV DNA polymerase. These mechanisms have been widely reviewed elsewhere. However, ACV had poor antiviral activity against HCMV [14]. Unlike HSV, HCMV does not express an enzyme with a specific TK activity. Thus, it was thought that the poor antiviral effects of ACV against HCMV stemmed from inefficient conversion of ACV from its pro-drug to drug form [14]. More recent works on ‘suicide gene therapy’ have demonstrated that ectopic expression of HSV TK can result in GCV phosphorylation [15], although this is likely due to the robust overexpression of HSV TK in those settings.

The guanine nucleoside analogue [9-[(1,3-dihydroxy-2-propoxy)methyl]guanine (DHPG)] was identified as an anti-HCMV compound in the early 1980s by the pharmaceutical company Syntex [16] and was shown to have anti-HCMV activity against HCMV and other herpesviruses such as HSV and VZV (but no obvious antiviral activity against another DNA virus, vaccinia virus, or an RNA virus, encephalomyocarditis virus) [14, 16]. Although DHPG was previously studied under other names, including 2′NDG and BW B759U, the generic name GCV is now used exclusively to describe this compound.

The use of GCV in humans came to the fore in the 1980s in compassionate use programmes for patients who had severe disease, for example, retinitis, associated with HCMV [10, 11]. Many of these individuals were subsequently understood to have advanced human immunodeficiency virus-related disease. As outlined in commentaries on that time period, including [11], the rapid development of GCV use in humans was not without regulatory challenges. For example, questions surrounding the ethics of conducting clinical trials wherein an effective drug (GCV) was withheld from a control group were of concern at the time. Due to these and other issues, a ‘dual-use’ policy was created wherein trials could be conducted while the drug was administered to those in need outside of the trial [11].

Clinical assessments of adults in the mid-1980s addressed the utility of GCV for treating life-threatening HCMV disease in immunocompromised hosts [10, 11]. Perhaps the most influential was the Collaborative DHPG Treatment Study Group [17]. This clinical trial evaluated GCV efficacy in 26 immunocompromised patients, 26 of whom had AIDS. This study demonstrated virologic suppression in 17/22 patients with HCMV disease confirmed by virologic assays. In this work, GCV was particularly effective for HCMV retinitis, improving disease in 11/13 patients, as well as for gastrointestinal disease, where 5/8 patients improved. However, this study identified limitations in the use of GCV against HCMV. GCV performed poorly in the treatment of HCMV pneumonia, where 4/7 patients died prior to completing therapy. Moreover, 79% of patients experienced reoccurrence (clinical or virologic) upon termination of therapy, suggesting that GCV provided suppression of HCMV instead of an absolute cure. This highlighted the necessity for the development of long-term GCV therapies, which were subsequently developed [8]. Nevertheless, following the establishment of GCV efficacy against HCMV, the US Food and Drug Administration (FDA) granted approval for the use of GCV (GCV sodium, sold as Cytovene) in 1989 for the treatment of HCMV retinitis in immunocompromised patients and the prevention of HCMV in transplant patients.

In adult human clinical trials of Cytovene, at least 83% of patients achieved a virologic response, with a 100-fold decrease in viral titres observed [18]. In patients with HCMV retinitis, intervention with GCV significantly extended the time to disease progression compared to untreated controls, with immediate intervention proving more successful. In transplant patients, GCV was equally successful, with a reduced incidence of HCMV disease from 43 to 16%. In bone marrow transplant patients, the incidence of HCMV disease fell to 3% compared with 43% in untreated patients. An oral GCV capsule was also utilized at high concentrations several times a day for maintenance treatment. However, it proved less successful than the IV treatment, which can be attributed to poor oral bioavailability of GCV. These data would later lead to the development of the orally bioavailable pro-drug valganciclovir (VGCV) [19].

## Analysis of GCV use in pre-clinical animal models and human clinical trials

Presently, GCV is the most widely used anti-HCMV drug in adults, for example some transplant recipients [5, 6]. As outlined elsewhere [5, 6], the use of GCV in adults is an increasingly clinically optimized area. However, GCV is not currently licenced for use during pregnancy, or for use in children. Therefore, GCV use in combating congenital HCMV infection is limited. To understand why this is, the data evaluating Cytovene in pre-clinical animal models must be understood. This data is alluded to, but not cited, in many diverse academic works on why GCV use is prohibited or restricted during pregnancy or in early life, including some works from our own laboratory (for example, [8]).

**Fig. 1.**
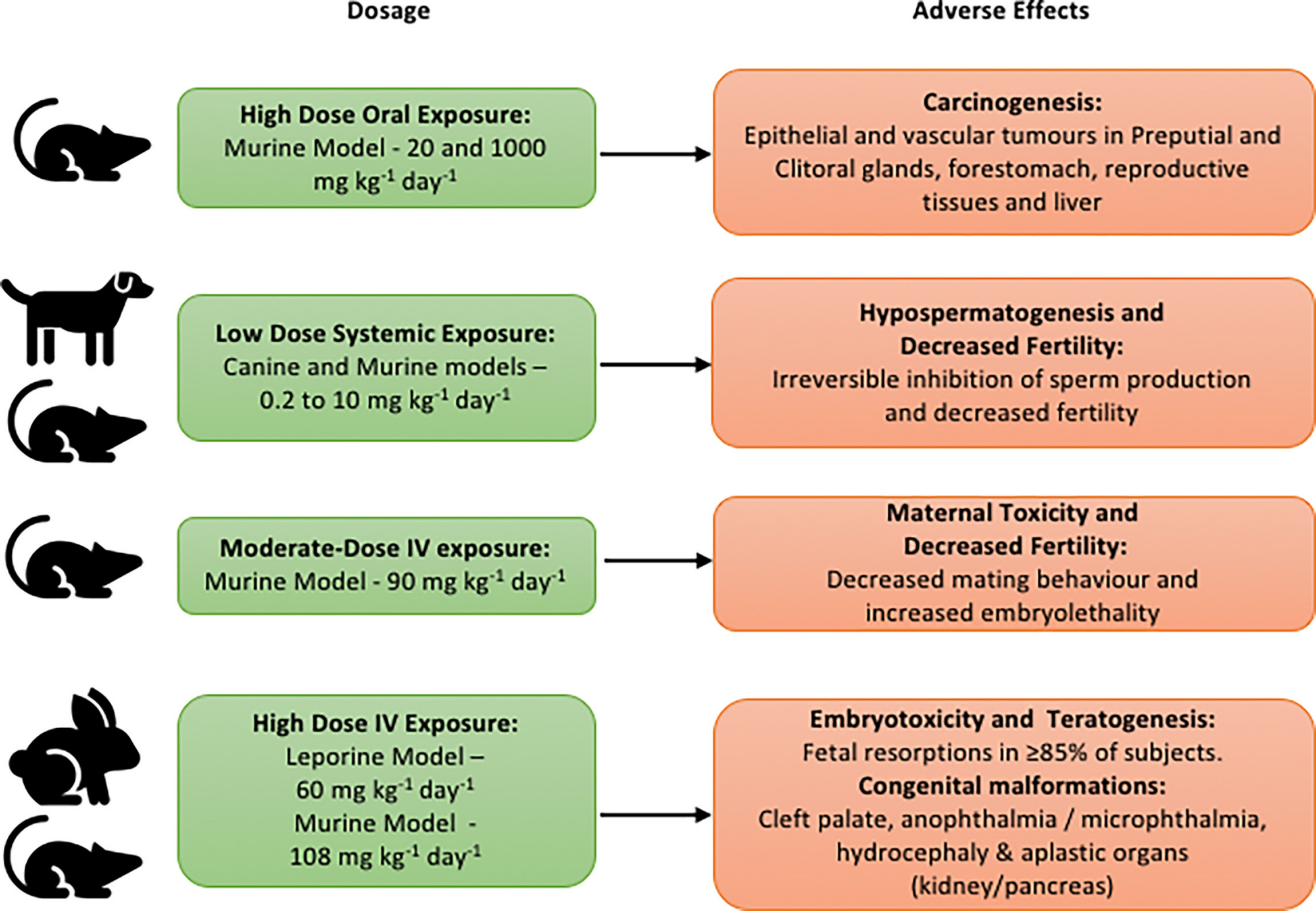
GCV (Cytovene) dosage and adverse effects in animal models. Dosage and adverse outcomes reported during pregnancy and development across uninfected *in vivo* mammalian models (canine, murine and leporine) following administration of GCV (Cytovene). Data from manufacturer’s prescribing information [18].

Overall, the use of Cytovene in uninfected animal models (HCMV does not replicate in non-human models) was defined by notable adverse effects during pregnancy and development (Fig. 1). These pointed to GCV’s potential for carcinogenesis, male infertility, maternal toxicity and teratogenicity in animal models and, for the most part, remain the basis for regulatory concern regarding long-term potential carcinogenic and reproductive toxicity associated with GCV during pregnancy or in children. This was underlined when in 1997 the California Office of Environmental Health Hazard Assessment (OEHHA) classified GCV as a chemical known to cause cancer and reproductive toxicity. This classification is currently reflected in the US FDA-approved labelling of GCV [9].

**Fig. 2.**
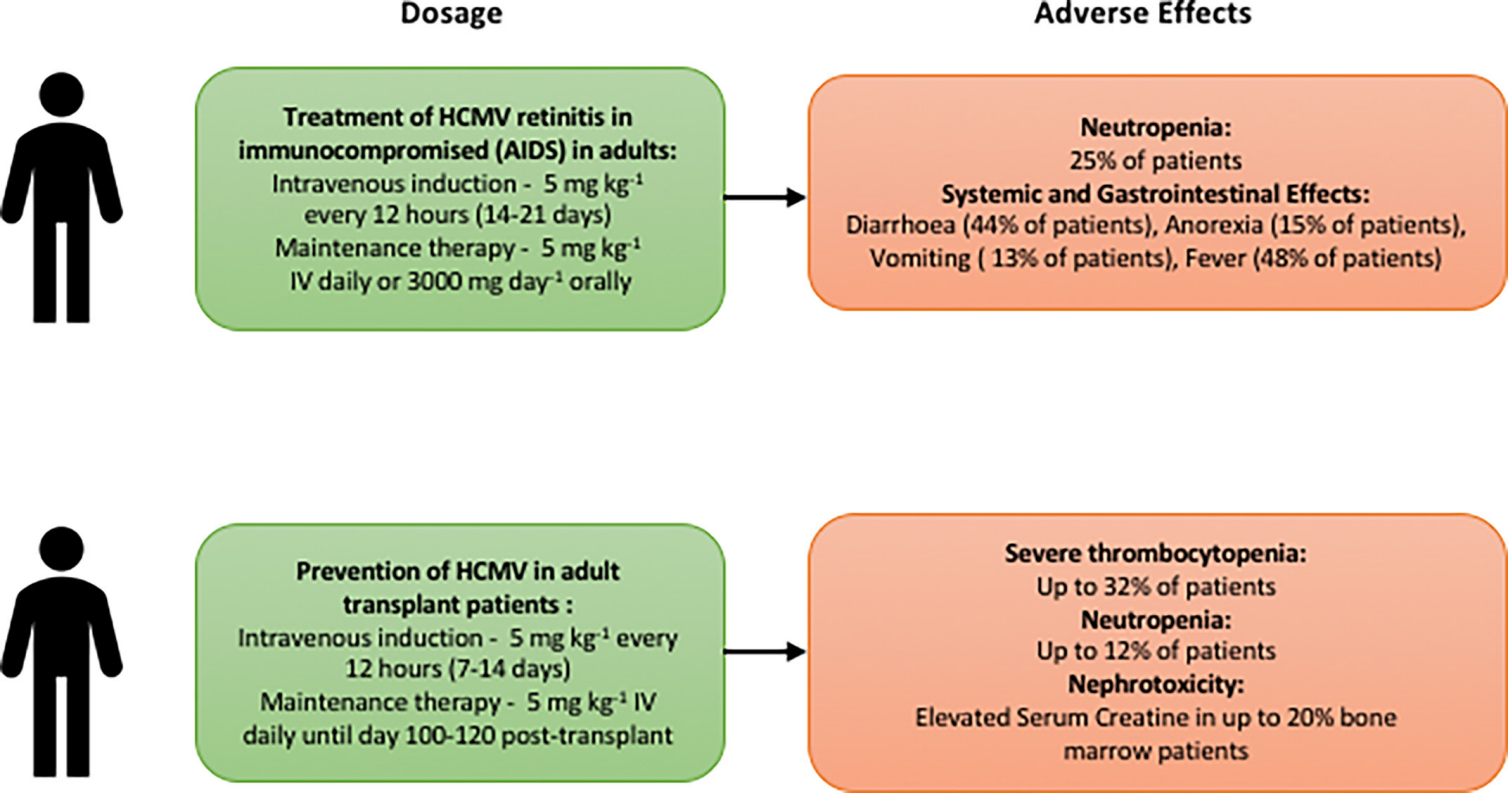
GCV (Cytovene) dosage and adverse effects in humans. Intravenous dosing protocols and their associated major adverse effects across immunocompromised HCMV patient populations (AIDS and transplant patients) following the administration of GCV. Data from manufacturer’s prescribing information [18].

It is, however, interesting to compare the data on Cytovene use in animal models (Fig. 1) and in HCMV-infected humans (Fig. 2). The adverse outcomes observed in animal models were largely observed at concentrations that exceeded the human dosages that had a therapeutic effect. Therefore, it remains possible that adverse effects in animals were due to effects of high dosage in these models and that few or no adverse effects that could complicate pregnancy or child development in humans would be observed if GCV was used at low concentrations. It also remains possible that adverse effects seen in animal models occur only in those animal models and not in humans. A possible reason for this would include the long-standing observation that human and non-human placentas are structurally different, and there is no guarantee that a molecule would be able to pass across the placenta to the foetus with equal efficiency in both human and non-human settings. These observations suggest that it could be possible to further develop GCV for use in various settings, including congenital infection, without observing the very serious adverse effects seen in animal models. Indeed, recent retrospective data analysis of GCV administration during pregnancy in humans [20] has reported that any adverse effects during pregnancy or at birth were not common when VGCV was administered during pregnancy and there was no increased reporting of adverse pregnancy outcomes or birth defects when VGCV was administered compared to valaciclovir (VACV), a drug not known to be associated with adverse effects. Efficacy of treatment in this study was not evaluated, and long-term follow-up of these children to examine long-term effects of GCV use during pregnancy was not pursued [20]. Moreover, recent data from a small-scale clinical trial administering VGCV during pregnancy has reported no abnormalities in any births during the trial [21]. However, expansion of this trial to include a greater number of participants is required before firm conclusions on GCV effects can be drawn. Additionally, efficacy of GCV treatment in the aforementioned trial was questionable as the majority of children born from HCMV seropositive mothers had detectable HCMV virus [21]. It is possible that the efficacy of treatment was poor due to low concentrations of GCV used, and further examination is required to understand the relationship between efficacy and any adverse effects.

Currently, GCV use to treat hearing loss in children with congenital HCMV infections is being trialled in various settings, and there are indications that GCV use can improve hearing. A prominent example of this was a large randomized, placebo-controlled trial comparing 6 weeks and 6 months GCV therapy in neonates with symptomatic congenital CMV disease. While there were no obvious differences in hearing improvement in the short term between groups, those neonates treated with GCV for 6 months had better long-term hearing outcomes than those treated for 6 weeks [22]. Moreover, those treated for 6 months had better developmental outcomes (including language acquisition and communication skills) than those treated for 6 weeks [22]. Further evaluation of any GCV adverse effects could be achieved by long-term monitoring of children treated with GCV in early life in studies such as that outlined above. However, this is currently problematic, as GCV is rarely used to treat HCMV disease in early life.

Therefore, the safety and efficacy of GCV use during pregnancy and early life to treat congenital infection are questions that require further study and data from animal models alone showing adverse effects in development should not preclude further investigation of these questions. However, a major finding of Cytovene use in humans was adverse effects such as neutropenia (Fig. 2), which cannot be overlooked. These effects likely involve the action of GCV on uninfected cells, as there is no evidence that HCMV can infect a wide range of haematopoietic lineage cells. Effects such as these can complicate GCV use in any patient population, including potential use to treat congenital HCMV disease. However, it is widely recognized that these effects are reversible upon suspension of GCV treatment, and at least one small-scale clinical trial has reported that neonatal neutropenia was not common when VGCV is administered during pregnancy [21].

It is possible that adverse effects in animal models and humans can come about via various mechanisms. Most likely these involve the incorporation of GCV into host cell DNA in uninfected cells. Therefore, to better understand how adverse effects come about the metabolism of GCV in infected and uninfected cells has to be understood. This can be gleaned from insights made after GCV came into clinical use, including recently presented data on GCV function, discussed below.

## Phosphorylation of GCV in infected and uninfected cells

The ability of GCV to cause adverse effects in uninfected animal models and in humans indicates that the GCV pro-drug can be converted to the GCV drug both in infected and uninfected cells. This has been demonstrated elsewhere. For example, pharmacokinetic analysis monitoring PBMCs from patients treated with GCV has demonstrated measurable accumulation of GCV-TP even in the absence of HCMV infection and directly correlated intracellular drug levels with haematologic toxicity [23].

**Fig. 3.**
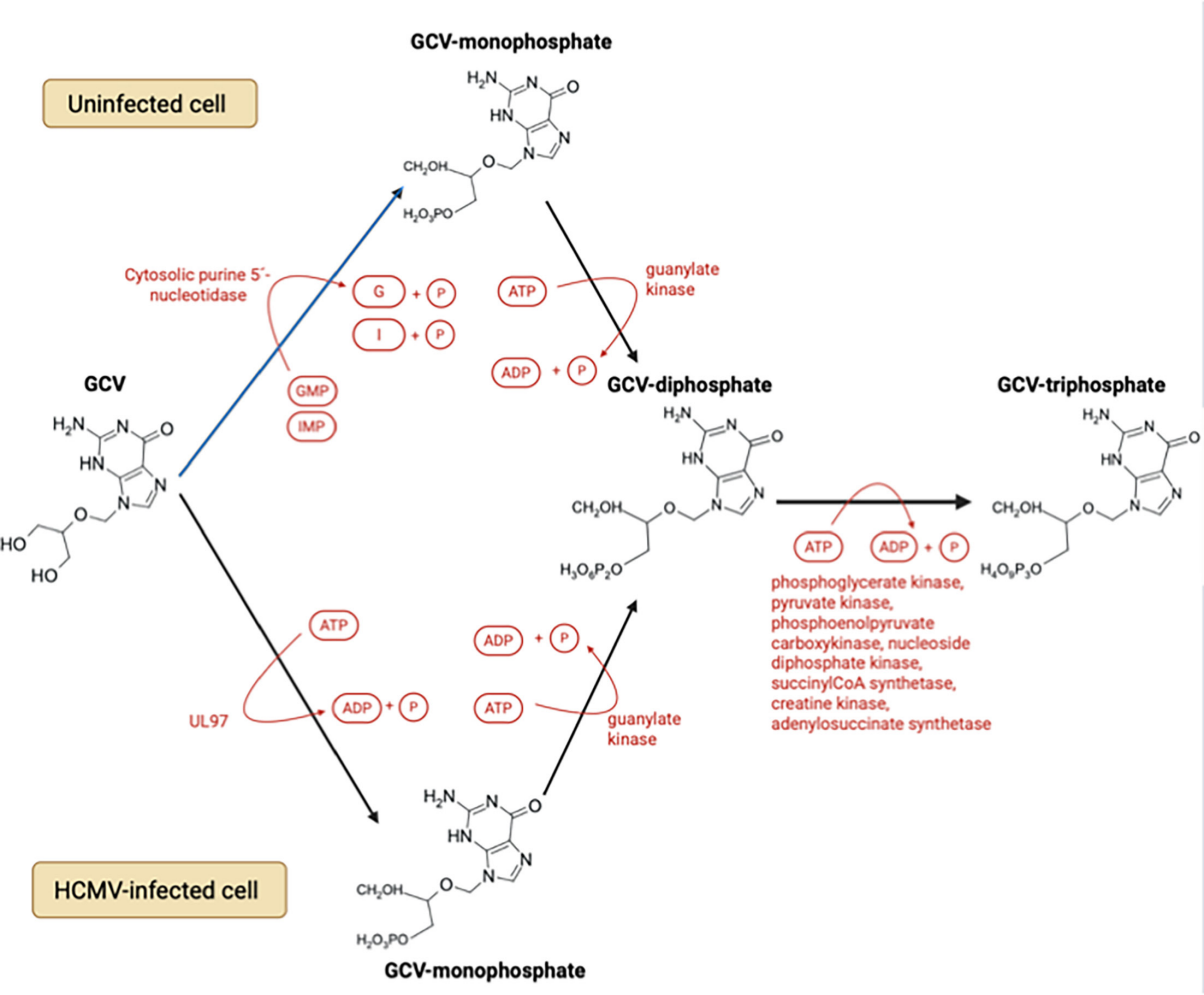
Phosphorylation of GCV to GCV-TP in uninfected and HCMV-infected cells. Differing phosphorylation of GCV to GCV-MP in uninfected (top) and HCMV-infected (bottom) cells is shown, as is phosphorylation of GCV-DP to GCV-TP. All phosphorylation steps, substrates and enzymes known or thought to be required at each step (see text) are highlighted in red. Created in https://BioRender.com. https://BioRender.com/jbt4a0l

GCV-TP is a nucleotide analogue of deoxyguanosine triphosphate (dGTP) that requires conversion from a monophosphate (MP) pro-drug to a TP form to exert its antiviral effect. In infected cells, the viral protein kinase UL97 phosphorylates GCV to its MP form (GCV-MP) [24], which is subsequently converted to a diphosphate (DP) moiety (GCV-DP) and then to the active TP (GCV-TP) by cellular kinases (Fig. 3). UL97 is a phosphotransferase that is functionally distinct from HSV TK and is capable of modifying both nucleosides and proteins. Analysis of GCV with purified guanylate kinase, a human cellular enzyme that regulates cellular GDP levels, has demonstrated that conversion of GCV-MP to DP can be mediated by guanylate kinase [25] (Fig. 3). However, it has not yet been demonstrated that guanylate kinase is responsible for GCV phosphorylation in HCMV-infected cells. It remains unclear what cellular enzymes are responsible for GCV-DP phosphorylation to GCV-TP. It has been demonstrated that purified versions of the cellular enzymes phosphoglycerate kinase, pyruvate kinase, phosphoenolpyruvate carboxykinase, nucleoside DP kinase, succinyl-CoA synthetase, creatine kinase and adenylosuccinate synthetase can phosphorylate ACV-DP [26]. Thus, it has been proposed [27] that these enzymes may also act to phosphorylate GCV-DP (Fig. 3). However, GCV-DP conversion to GCV-TP by any of these aforementioned enzymes in HCMV-infected cells has yet to be demonstrated.

Interestingly, recent data has demonstrated that conversion of GCV to GCV-TP is reversible, wherein the cellular enzyme nucleoside DP-linked moiety X-type motif 15 (NUDT15) DP can convert GCV-TP to GCV-MP [28, 29]. As demonstrated by studies of patients carrying NUDT15 polymorphisms [28] or by assaying HCMV replication in the presence of GCV and the NUDT15 inhibitor TH8321 [29], loss of NUDT15 function can potentiate the effects of GCV against HCMV *in vivo* and *in vitro* [28, 29].

**Fig. 4.**
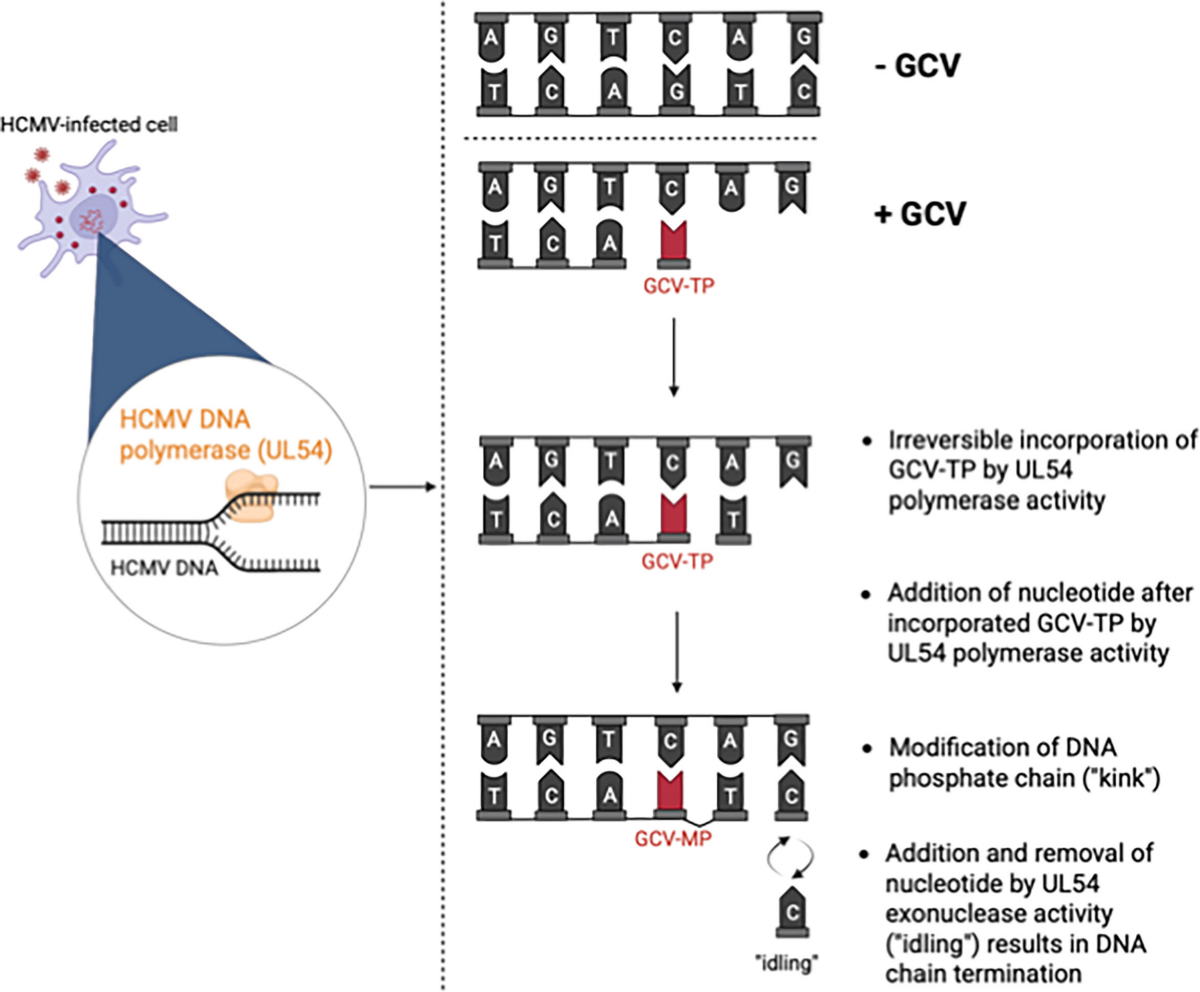
Inhibition of HCMV DNA replication by GCV. The absence of GCV in the HCMV-infected cell (-GCV) has no effect on HCMV DNA replication. As described elsewhere [30], the presence of GCV in the HCMV-infected cell (+GCV) leads to GCV-TP incorporation into HCMV DNA by the HCMV DNA polymerase UL54. The mechanism by which HCMV DNA chain elongation is terminated is described in the figure and the text. Created in https://BioRender.com. https://BioRender.com/m8sr8ot

In HCMV-infected cells, GCV-TP competes with dGTP for incorporation into nascent viral DNA synthesized by the HCMV polymerase catalytic subunit UL54. The molecular basis of DNA synthesis inhibition by GCV has only been uncovered in the recent past [30] (Fig. 4). Unlike ACV, GCV consists of a guanine linked to an acyclic moiety that lacks the equivalent 2′-position but contains a 3′-hydroxyl group. This promotes GCV incorporation into the viral DNA chain via a mechanism distinct from that of ACV. Incorporation of GCV-TP into HCMV DNA likely induces a conformational distortion (described as a ‘kink’) in the DNA phosphate backbone that prevents the further translocation of UL54 along DNA (Fig. 4). This process traps UL54 in a state wherein UL54 polymerase activity can add the next nucleotide after GCV-TP, but the conformational distortion caused by GCV-TP incorporation causes UL54 exonuclease activity to remove any subsequent nucleotide incorporated into DNA (Fig. 4). Thus, the cycle of addition and removal of a nucleotide downstream of GCV-TP incorporation into DNA (described as polymerase ‘idling’) prevents HCMV DNA chain elongation by the HCMV DNA polymerase and, therefore, genome replication (Fig. 4). Resistance to GCV can come about via mutation in either UL54 or UL97 [31]. HCMV drug resistance mutations have been previously reviewed [31]. However, HCMV drug resistance, in general, has not been recently reviewed, and a more up-to-date review would possibly be helpful to various colleagues.

Like GCV phosphorylation in HCMV-infected cells, the mechanism by which phosphorylation of GCV occurs in uninfected cells is also not well understood. In the absence of UL97, a cellular protein must be responsible for phosphorylation of GCV to GCV-MP. As yet, it is unknown what cellular enzyme is responsible for the production of GCV-MP in uninfected cells. However, evidence points toward cytosolic purine 5′-nucleotidase as a candidate enzyme (Fig. 3), as it has been reported that purified rat cytosolic purine 5′-nucleotidase can mediate phosphorylation of ACV to ACV-MP [32]. Though cytosolic purine 5′-nucleotidase has been shown to primarily act as a hydrolase, it can act as a phosphotransferase in the presence of high concentrations of phosphate donors, such as inosine monophosphate (IMP) or guanosine monophosphate (Fig. 3). This mechanism creates a phospho-enzyme intermediate, which then allows the enzyme to transfer a phosphate group to nucleosides, such as guanosine [33]. Based on these data, it is possible that cytosolic purine 5′-nucleotidase acts to phosphorylate GCV, thus forming GCV-MP. Consistent with this hypothesis, drugs that promote accumulation of IMP also promote phosphorylation of GCV in uninfected cells [34]. However, it has not yet been demonstrated that cytosolic purine 5′-nucleotidase is responsible for GCV phosphorylation in uninfected cells.

Following monophosphorylation, the cellular kinases responsible for converting GCV-MP to GCV-DP to GCV-TP in HCMV-infected cells discussed above likely also mediate GCV-MP to GCV-DP to GCV-TP conversion in uninfected cells (Fig. 3). It remains unknown what cellular DNA polymerase enzymes could be responsible for incorporating GCV-TP into cellular DNA. But, such is the structural similarity between GCV-TP and dGTP, there should be no obvious restriction to cellular DNA polymerase enzymes recognizing GCV-TP.

The effects of GCV incorporation into cellular DNA during HCMV treatment have not been rigorously investigated. However, analysis of so-called ‘suicide gene therapy’ approaches (ectopic expression of HSV TK in the presence of GCV, usually in cancer cells) provides some indication of GCV action. Overall, it is thought that incorporation of GCV into cellular DNA in those settings results in DNA single-stranded breaks [35], which result in p53-associated mechanisms such as the translocation of pro-apoptotic factors, for example CD95, to the cell surface [15]. It is possible that other mechanisms occur in a range of different cancer cells or that these mechanisms may be restricted to cancer cells. However, these data provide a model for how GCV may function during the adverse effects that we discuss above.

While many aspects of the data outlined above require clarification and confirmation, these data point toward how GCV-TP can be found in both HCMV-infected and uninfected cells. However, it is likely that the conversion of GCV to GCV-MP by UL97 is considerably more efficient than the conversion of GCV to GCV-MP by cellular enzymes. For example, it has been demonstrated that GCV-TP accumulated in uninfected cells at levels approximately tenfold lower than in HCMV-infected cells [23].

Overall, these data demonstrate that, while inefficient, conversion of GCV to GCV-TP in uninfected cells is possible. While the data described above was derived from the study of human proteins, homologues or orthologues of those proteins are present in non-human cells. Therefore, the incorporation of GCV-TP into cellular DNA is likely the cause of adverse effects seen in humans and animal models. It remains unknown if the full repertoire of cellular enzymes capable of promoting GCV metabolism in infected or uninfected cells has been identified, or how the function of cellular enzymes could be modified or inhibited so as to prevent metabolism of GCV in uninfected cells. This possibility seems unlikely as the cellular enzymes discussed above are very likely essential for cell function under physiological conditions, and inhibiting their function would itself result in adverse effects. Instead, it is more likely that to avoid adverse effects of GCV, new strategies to the use of GCV or alternatives to the use of GCV could be considered.

## New GCV strategies and alternatives to GCV

There have been strategies to improve GCV efficacy against HCMV, which would allow lower and shorter dosages of the drug to be administered. These have involved the use of a clinically used immunosuppressive drug mycophenolate mofetil to lower cellular nucleotide pools and ensure that GCV-TP is always in excess compared to cellular nucleotides in infected cells. This produces strong anti-HCMV effects in the laboratory [36, 37]. However, the use of an immunosuppressive drug during pregnancy is not advisable.

Similarly, the use of the NUDT15 inhibitor TH8321 has the ability to enhance the antiviral effects of GCV [29]. To our knowledge, loss of NUDT15 has no obvious adverse effects. Loss of NUDT15 is not immunosuppressive and has no obvious pathology, other than sensitivity to thiopurine drugs [28]. Plus, treatment of cells with the NUDT15 inhibitor TH8321 had no obvious effect on cell viability [29]. At present, TH8321 is only regarded as a chemical probe for NUDT15 function [29], and its efficacy would have to be improved to achieve therapeutic effects. Thus, pre-clinical development of TH8321 is required before it could be used as a therapeutic.

A strategy that is as yet largely unexplored is the use of combination therapy in humans against HCMV. Currently, GCV is used as a monotherapy. There should be no barrier to using GCV in combination with other antiviral drugs that have different targets [7, 8]. This would likely allow the use of low doses of GCV and may avoid adverse effects of GCV that we discuss above. This strategy could include many of the anti-HCMV drugs discussed below.

As an alternative to GCV use, other direct-acting antivirals against HCMV have been developed [1, 7, 8]. The non-nucleoside UL54 inhibitor foscarnet can currently be used against GCV-resistant HCMV viruses, but the well-known toxicity of this drug prevents its use in a range of settings, including inhibiting congenital infection. The UL97 inhibitor maribavir and the HCMV DNA packaging protein UL56 inhibitor letermovir have been used against HCMV in adult clinical settings. However, they also have shortcomings such as lack of clinical efficacy or the development of drug resistance mutations. Long-standing data has shown that various other nucleoside analogues, for example, those that have been used in the early days of cancer therapy, have strong anti-HCMV activity [38]. However, they have well-known toxic effects, and many of them are not used in humans today. Various attempts to inhibit the function of HCMV-encoded proteins other than those mentioned above are ongoing in multiple laboratories. However, no compounds derived from these studies appear ready for further clinical development.

Perhaps the most obvious alternative to GCV is the recent development of VACV, an orally bioavailable form of ACV, as an anti-HCMV drug. To date, VACV use has largely been restricted to the treatment of HSV, as VACV has poor anti-HCMV effects compared to GCV [39]. However, developing clinical studies indicate that high-dose VACV has the potential to effectively prevent vertical HCMV transmission during pregnancy with little or no adverse effects [40]. The clinical use of VACV in pregnancy requires future development, including assessment of large trial groups and long-term follow-up of children born from pregnancies where VACV was used. However, it would be very useful to understand VACV use against HCMV in other settings, including early life and adult settings.

We and others have argued that host-directed therapy targeting cellular factors required for HCMV replication should be further developed as an alternative to GCV use. We have recently reviewed this area [8]. This would promote the development of compounds such as artemisinin compounds, kinase inhibitors and many others, which have the potential to be used without adverse effects in both adult and child populations.

## Conclusions and final thoughts

First and foremost, the longevity of GCV is very unusual in the field of antiviral chemotherapy, where it is common that second- or third-generation drug regimens are in routine use against many human viruses. This unusual longevity of GCV is especially noteworthy as, given its effects on animal models, it is arguable that GCV may not have been approved for use in humans, if it were being developed today. The widespread use of GCV in adults is a somewhat mature area, whereas the use of GCV to treat congenital infection remains in development.

The study of GCV in animal models has, rightly, promoted a great deal of caution toward the use of GCV during pregnancy and early life. However, development of further clinical data (including retrospective analysis of treatment and prospective randomized control trials) may yet demonstrate that GCV can be used, perhaps with limited efficacy, without obvious adverse effects to treat congenital infection. In the first instance, this would likely focus on administration of GCV during pregnancy, as this is an area where developing data suggests there could be few or no adverse effects in the presence of GCV. These studies could then be expanded to the treatment of symptomatic neonates with GCV.

Adverse effects of GCV likely involve the incorporation of the drug into DNA within uninfected cells, which requires conversion of the GCV pro-drug to the drug. It is remarkable how little is known about each GCV conversion step in both uninfected and infected cells, and the ability of cellular enzymes to mediate these steps in both uninfected and infected cells is yet to be demonstrated.

It remains unclear if the adverse effects of GCV, brought about by the incorporation of GCV-TP into uninfected cells, can be mitigated. Therefore, it may be necessary to seek strategies and alternatives to the use of GCV outlined here.

## Abbreviations

ACV: aciclovir
dGTP: deoxyguanosine triphosphate
DHPG: 9-[(1,3-dihydroxy-2-propoxy)methyl]guanine
DP: diphosphate
FDA: Food and Drug Administration
GCV: ganciclovir
HCMV: human cytomegalovirus
HSV: herpes simplex virus
IMP: inosine monophosphate
MP: monophosphate
TK: thymidine kinase
TP: triphosphate
VACV: valaciclovir
VGCV: valganciclovir
VZV: varicella-zoster virus
